# Longitudinal Changes in Thyroid Hormones and Serum Albumin Following CABG, AVR, and PCI: An Exploratory Electronic Medical Record Study

**DOI:** 10.3390/medsci14020196

**Published:** 2026-04-14

**Authors:** Pareek Aayushi, Hossam Gad, Abdelhamed Elgazar, Krzysztof Laudanski

**Affiliations:** 1 Department of Critical Care Medicine, Mayo Clinic, Jacksonville, FL 32224, USA; aayushipareek904@gmail.com; 2 Department of Anesthesiology and Perioperative Care, Mayo Clinic, Rochester, MN 55902, USA; gadhossam.a@gmail.com (H.G.); elgazar.abdelhamed@mayo.edu (A.E.)

**Keywords:** surgery, long-term effects, thyroid hormones, T3, T4, TSH, inflammation, albumin, electronic medical records

## Abstract

Background: Surgical trauma disrupts hormone networks, but the duration required for these systems to recover remains unclear. We hypothesize that significant perioperative stress would trigger protracted abnormalities of the thyroid axis extending past 28 days. Methods: This retrospective exploratory study analyzed opportunistically obtained thyroid-related laboratory values (free T3 [FT3], free T4 [FT4], and thyroid-stimulating hormone [TSH]) and serum albumin from electronic medical records of patients undergoing CABG, AVR, or PCI between 2017 and 2022. Preprocedural baseline values were compared with post-procedural serum levels measured during the acute peri-procedural period (0–30 days), early recovery (31–90 days), intermediate recovery (91–180 days), late recovery (181–365 days), medium-term follow-up (1–2 years), and long-term follow-up (>2 years). Results: Free T3 demonstrated early suppression across all procedures, most pronounced in CABG during the acute peri-procedural period, with partial recovery at later timepoints. AVR showed moderate suppression at early and long-term follow-up, while PCI demonstrated minimal and inconsistent changes. Free T4 remained relatively stable across procedures, with limited significant post hoc differences after adjustment. TSH showed significant temporal variability in CABG and AVR but not in PCI. Serum albumin demonstrated marked early decline, most pronounced in CABG, with partial recovery over time, whereas AVR showed delayed long-term suppression. Data availability declined substantially at later timepoints across all biomarkers. Conclusions: In this retrospective exploratory analysis, CABG was associated with the most pronounced early perturbations in thyroid and albumin trajectories, while PCI and AVR demonstrated more heterogeneous temporal patterns. These findings are hypothesis-generating and should be interpreted cautiously given non-protocolized laboratory follow-up, substantial missingness, and potential selection bias.

## 1. Introduction

Surgical trauma disrupts homeostasis, affecting the immune system, metabolism, and hormone networks. The duration required for these systems to recover remains unclear. Some also suggest that more severe stress will result in more profound disturbances, but no definite evidence was presented [1,2]. If the disruption in homeostasis persists, the long-term benefit of surgery may be curtailed by the pathological influence of post-surgical allostasis disorder [1,3,4,5].

Thyroid hormones are essential for maintaining various aspects of homeostasis, including metabolism, immune system function, tissue repair, and cognitive function [6]. Thyroid-stimulating hormone (TSH) regulates the release of T3 and T4, and the metabolism of these hormones is essential in determining the tissue-specific effects of the thyroid axis [6,7,8,9]. Given this, it is not surprising that surgery can disrupt the thyroid axis [1,10,11,12]. For instance, Sabatino et al. found that patients undergoing CABG experienced significant declines in serum total triiodothyronine (T3) and free T3 (FT3) concentrations during and six days postoperatively, which remained low for several days [11]. Others demonstrated similar findings [10,12]. These disruptions have been shown to persist for several days, and some of these abnormalities are referred to as nonthyroidal illness syndrome and may persist for up to 6 months when peri-surgical thyroid disturbances occur [12]. However, it has not been defined when peri-surgical thyroid disturbances are fully resolved. Though short-term effects are often considered, the long-term impact of protracted changes is significantly less explored. In case of patients diagnosed with coronary artery disease, being both a metabolic and immune phenomenon, the disruption of the thyroid may accelerate the progression of the disease [2,10,13]. Therefore, it can be hypothesized that if thyroid abnormalities persist long-term after surgery, their presence may limit the benefits of the surgery itself.

Our study aims to explore the dynamics of the thyroid axis after surgery. Our overall hypothesis was that perioperative stress would trigger protracted abnormalities of the thyroid hormone axis extending past the traditional time of recovery of 28 days. Furthermore, we hypothesize that more severe stressors will trigger profound abnormalities in the thyroid axis by investigating the post-procedure abnormalities after percutaneous coronary intervention (PCI) versus much more taxing surgical procedures like coronary artery bypass graft (CABG) or aortic valve replacement (AVR) surgery. Since our study is exploratory, we used a previously established methodology to identify lab values pertinent to the hormonal axes under study [14,15]. Finally, we presumed that hormonal abnormalities would persist despite resolution of the active post-surgical inflammatory process, as signified by reversal of the post-surgical catabolic process (albumin).

## 2. Materials and Methods

### 2.1. Ethical Considerations

The University of Pennsylvania’s Institutional Review Board (Philadelphia, PA, USA) approved this retrospective study (#834697).

### 2.2. Study Design

This is a feasibility and exploratory study that employs a retrospective review of existing data extracted from electronic medical records (EMRs) to identify opportunistic lab values collected during routine care. Considering the pilot nature of the study, we did not perform a power analysis.

### 2.3. Study Analysis Procedures

**Figure 1:**
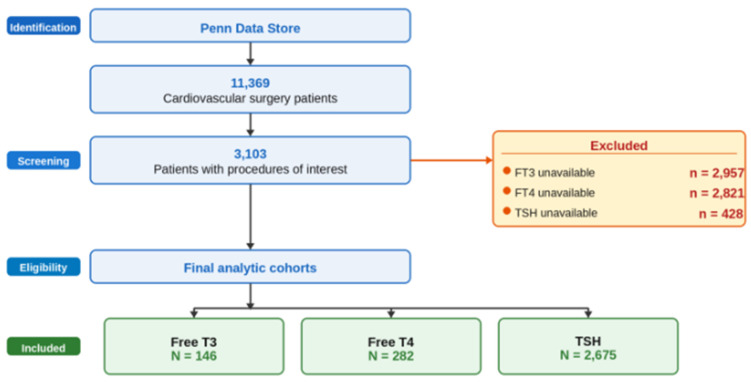
Strobe flow diagram. T3—triodothyronine; T3—thyroxine; TSH—thyroid-stimulating hormone; n—subgroup count.

Electronic medical records (EMRs) were used to collect all surgeries coded as coronary artery bypass graft (CABG; ICD-10 codes: 021009W, 02100AW, 02100Z9, 021109W, 021209W, and 02100Z8), and percutaneous coronary interventions (PCI; ICD-10 code: 027034Z) as two interventions to improve atherosclerosis progression with dramatically different inflammatory burden. Surgery for aortic valve replacement (AVR; ICD-10 codes: 02RF0JZ and 02RF08Z) was used as a control for invasive surgical interventions, but was conducted mostly due to degenerative illness, not CAD. The database was processed using Python 3.14.4 software. Figure 1 depicts the data flow. Table 1 shows individual lab cohort sizes.

Patient records were reviewed and assessed for thyroid abnormalities (Free T3, Free T4, and TSH). Thyroid laboratory values were categorized into predefined temporal windows relative to the procedural date. Baseline was defined as the most recent laboratory value collected within 90 days prior to the procedure. After surgery, we allocated variables to the following time bins: acute peri-procedural period (0–30 days after procedure), early recovery (31–90 days), intermediate recovery (91–180 days), late recovery (181–365 days), medium-term follow-up (1–2 years), and long-term follow-up (>2 years) [15]. If multiple laboratory measurements were available within a given time window, the mean value was used for analysis.

### 2.4. Statistical Analysis

To account for inter-laboratory variation in assay reference ranges, thyroid laboratory values, and albumin, where applicable, were transformed into Z-scores to facilitate inter-laboratory comparison across varying assay reference ranges. Raw laboratory values were recorded in standard clinical units (FT3: pg/mL, FT4: ng/dL, TSH: µIU/mL, and albumin: g/dL) and subsequently standardized as Z-scores for comparative analyses across laboratories. Distributional assumptions were assessed using the Shapiro–Wilk test and visual inspection of histograms and distribution plots. Because several subgroup-timepoint strata demonstrated non-normal distributions, non-parametric methods were used where appropriate.

Continuous variables are presented as mean ± standard deviation (SD) or median and interquartile range (IQR), as appropriate. Overall, within-group temporal differences were assessed using the Kruskal–Wallis test. Post hoc comparisons versus baseline were performed using the Wilcoxon signed-rank test when sufficient paired observations were available. Selected between-group comparisons at individual timepoints were assessed using the Mann–Whitney U test. Both raw and multiplicity-adjusted *p*-values were examined for post hoc interpretation, including Bonferroni correction. Effect sizes were summarized using Cohen’s d and interpreted conventionally as small (0.2), moderate (0.5), and large (0.8) [16,17]. Statistical significance was defined as a two-sided *p*-value < 0.05.

Temporal trends were visualized using Python-generated boxplots showing the median, interquartile range, and whiskers extending to 1.5 × IQR. All analyses and visualizations were performed using Python 3.13 with SciPy, Pandas, Matplotlib, and Seaborn [16,17,18].

## 3. Results

### 3.1. Characteristics of the Sample

Separate datasets were analyzed for each biomarker (free T3, free T4, and TSH) (Table 2). Baseline characteristics differed across procedural cohorts, particularly in the prevalence of atrial fibrillation and diabetes, reflecting underlying differences in cardiovascular disease populations undergoing each intervention. Race distribution was similar across groups in the Free T3 cohort (*p* = 0.85) but differed significantly in the Free T4 and TSH cohorts (both *p* < 0.001), with a higher proportion of African American patients in the PCI group.

### 3.2. Changes in Thyroid Axis Hormones After Cardiac Procedures

**Figure 2:**
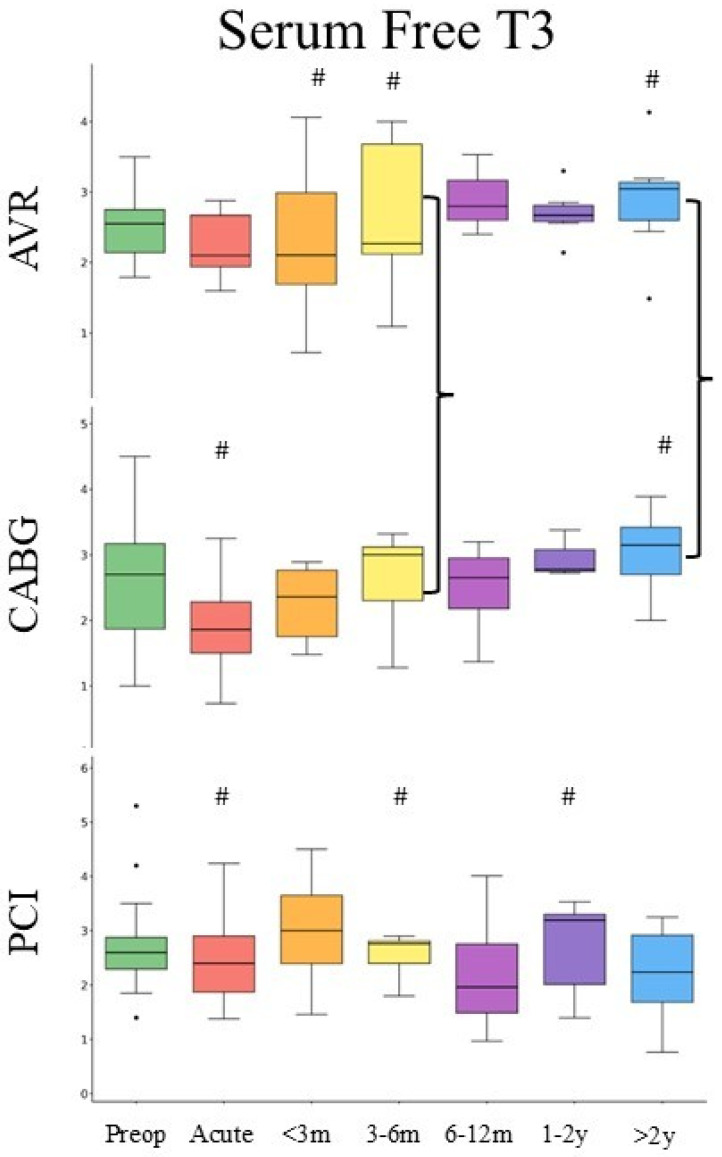
Changes in serum FT3 after AVR, CABG, and PCI. Boxplots display the median (central line), interquartile range (box), and whiskers extending to 1.5 × IQR. # denotes post hoc comparison versus baseline (*p* < 0.05). Brackets show the difference between CABG and AVR in the same time window. Serum FT3 is presented as pg/mL.

A total of 146 patients with free T3 (FT3) were available (AVR: *n* = 44, CABG: *n* = 48, and PCI: *n* = 54) (Table 1). Mean baseline FT3 levels across all groups were 2.69 ± 1.56 [pg/mL], with no significant differences between groups. FT3 levels declined across all procedures during the acute peri-procedural period, with the most pronounced reduction observed in the CABG cohort (d = 0.89). This suppression persisted into the early recovery phase (31–90 days) with a moderate effect size (d = 0.48), followed by a gradual return toward baseline at later timepoints. AVR demonstrated moderate suppression during the acute peri-procedural period (d = 0.60) and at long-term follow-up (>2 years), whereas PCI showed minimal and inconsistent changes over time (d = 0.23). Overall temporal variation in FT3 was observed in the CABG cohort (*p* = 0.03), while AVR and PCI did not demonstrate significant longitudinal variation (Figure 2). However, pairwise comparisons versus baseline did not remain statistically significant after adjustment.

**Figure 3:**
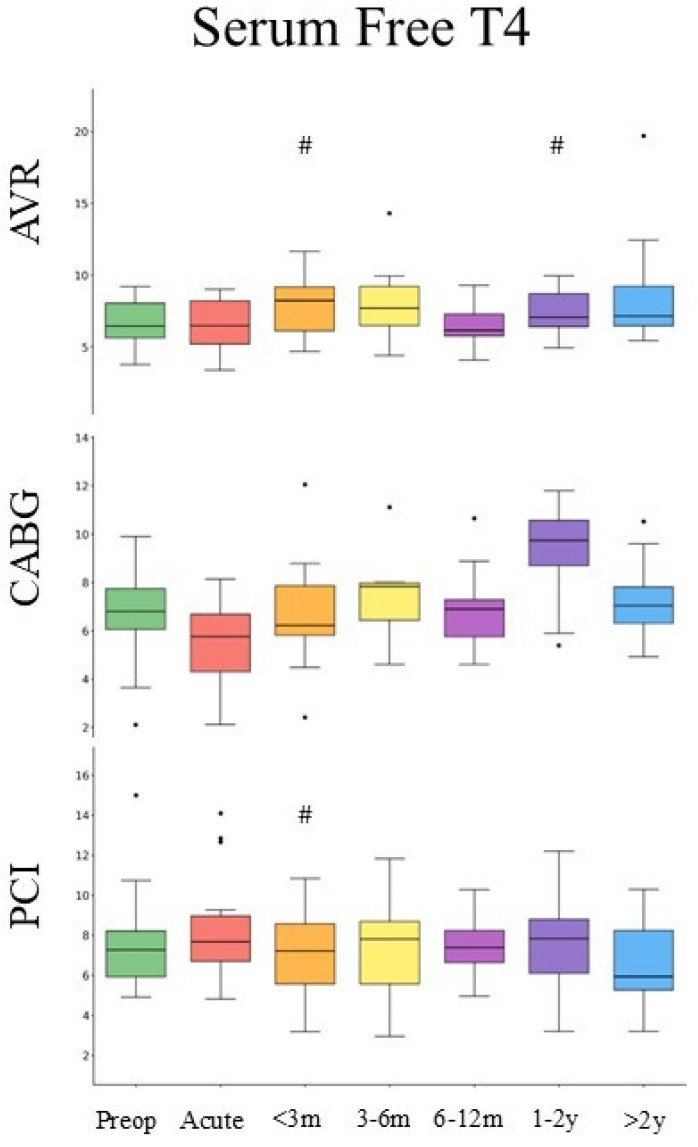
Changes in serum FT4 after AVR, CABG, and PCI. Boxplots display the median (central line), interquartile range (box), and whiskers extending to 1.5 × IQR. # denotes post hoc comparison versus baseline (*p* < 0.05). Brackets show the difference between CABG and AVR in the same time window. Serum FT4 is presented as ng/dL.

FT4 measurements were available in 282 patients (AVR: *n* = 84, CABG: *n* = 92, PCI: *n* = 106) (Table 1), with comparable baseline values. Baseline mean serum fT4 was 7.33 ± 2.24 [pmol/L] and did not differ across the patient populations studied. Free T4 levels showed subtle procedural differences: at acute peri-procedural and early recovery (d = −0.65 for AVR; d = 0.01 for CABG; and d = 0.22 for PCI), whereas at long-term follow-up, values remained moderately suppressed or returned to baseline depending on the intervention (Figure 3). Only CABG showed statistically significant temporal variability (Kruskal–Wallis *p* = 0.016), whereas AVR and PCI showed no significant overall change. No pairwise comparisons met significance after correction for multiple testing.

**Figure 4:**
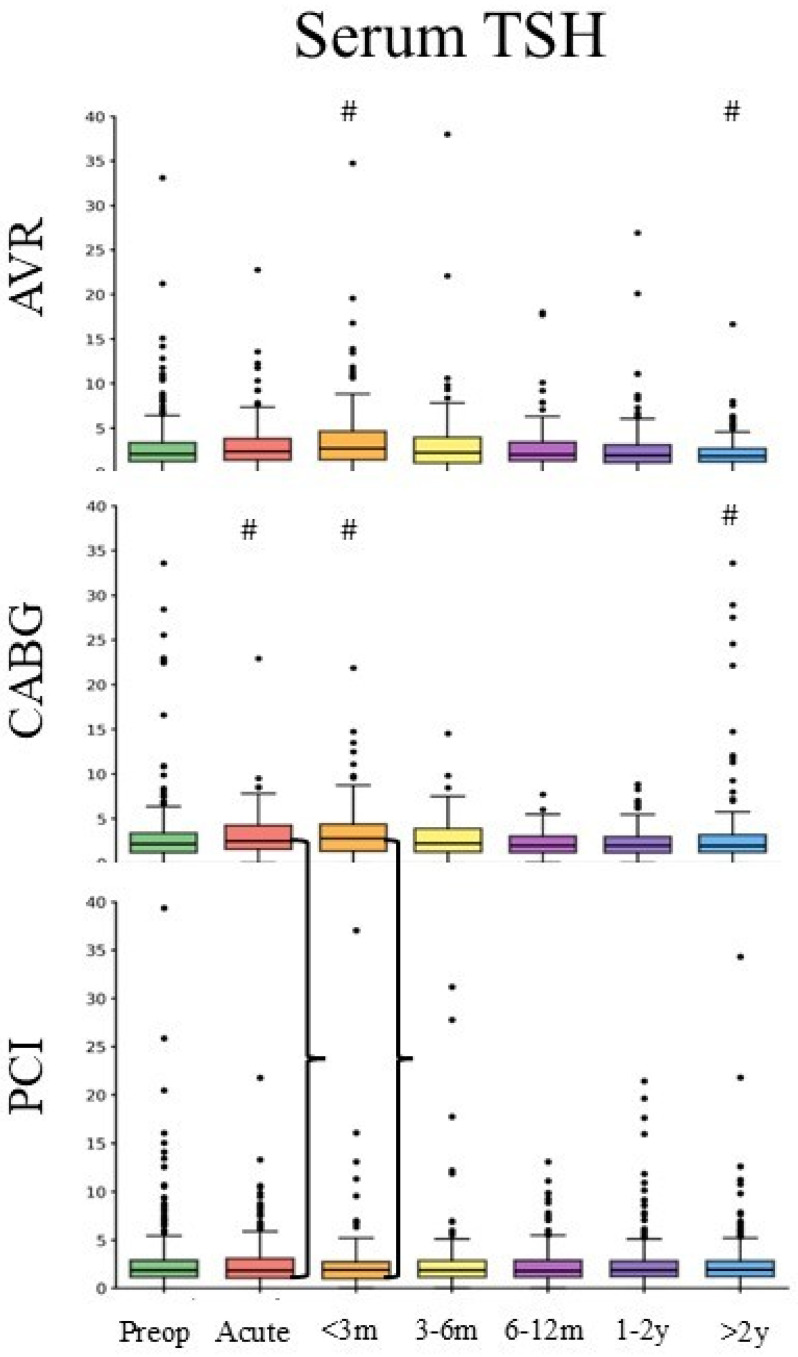
Changes in serum TSH after AVR, CABG, and PCI. Boxplots display the median (central line), interquartile range (box), and whiskers extending to 1.5 × IQR. # denotes post hoc comparison versus baseline (*p* < 0.05). Brackets show the difference between CABG and AVR in the same time window. Serum TSH is presented as µIU/mL.

TSH measurements were available in 2675 patients (AVR: *n* = 681, CABG: *n* = 824, and PCI: *n* = 1170) (Table 1). Baseline serum TSH was 3.16 ± 6.81 µIU/mL, and there was no difference between the groups. TSH levels increased during the acute peri-procedural and early recovery periods (d = −0.27 for AVR; 0.02 for CABG; and −0.04 for PCI), followed by a decline toward or below baseline at later timepoints (d = −0.008 for AVR; 0.09 for CABG; and −0.09 for PCI) (Figure 4). Both AVR (*p* = 0.003) and CABG (*p* = 0.0001) demonstrated significant TSH variability over time, with CABG showing early increases that did not persist beyond the intermediate recovery period. PCI did not show a significant temporal change. Pairwise comparisons at acute and early recovery time points revealed significant differences between PCI and AVR (*p* = 0.01 and 0.003, respectively), indicating earlier and more pronounced TSH perturbation following surgical AVR.

**Figure 5:**
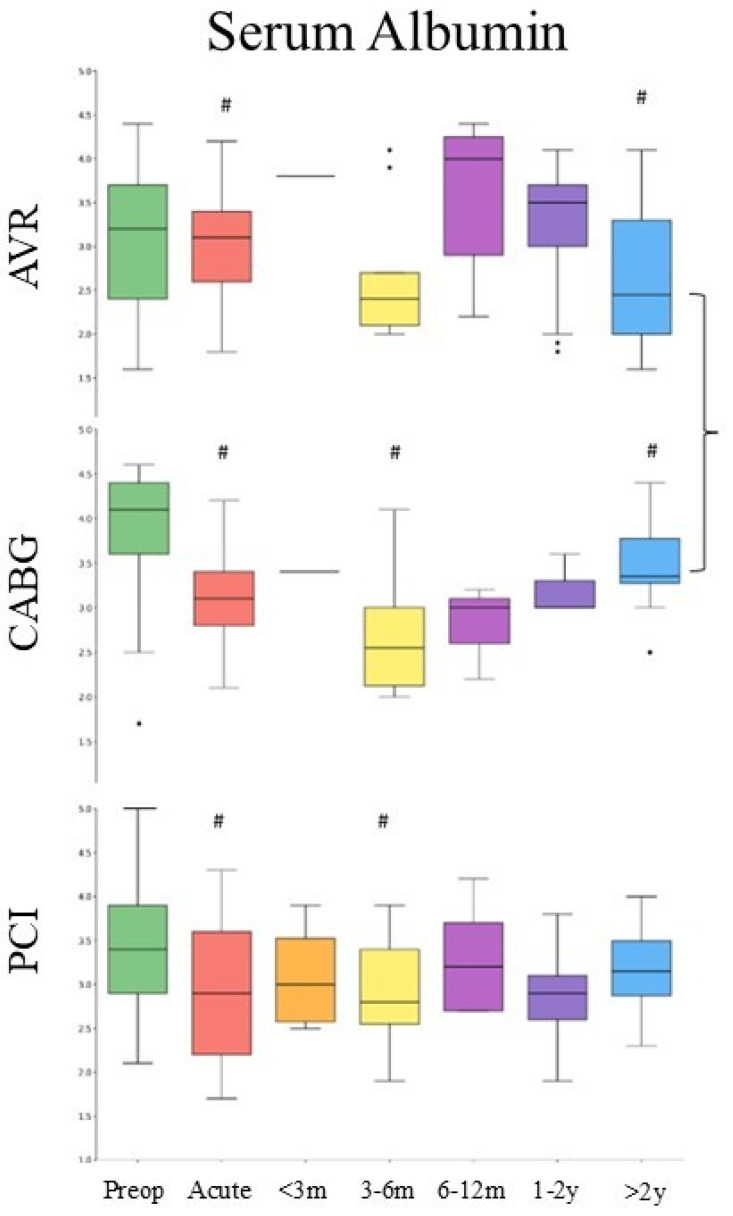
Changes in serum albumin after AVR, CABG, and PCI. Boxplots display the median (central line), interquartile range (box), and whiskers extending to 1.5 × IQR. # denotes post hoc comparison versus baseline (*p* < 0.05). Brackets show the difference between CABG and AVR in the same time window. Serum albumin is presented as g/dL.

Significant temporal variation in serum albumin levels was observed across all procedures, with the most pronounced changes in the CABG cohort (Figure 5). In CABG patients, albumin levels declined during the acute peri-procedural period (3.09 ± 0.43 g/dL) and reached a nadir during intermediate recovery (91–180 days; 2.66 ± 0.66 g/dL), representing a significant reduction compared to baseline (Wilcoxon *p* = 0.002; Cohen’s d = 0.88). Partial recovery was observed at long-term follow-up (>2 years; 3.48 ± 0.52 g/dL), although levels remained lower than baseline (*p* = 0.03; d = 0.83). In the AVR cohort, albumin levels showed minimal change during the acute peri-procedural period but demonstrated significant suppression at long-term follow-up (>2 years; *p* = 0.0031; d = 1.07). Overall temporal variation was significant (Kruskal–Wallis *p* = 0.005), although most post hoc comparisons were limited by small sample sizes. In PCI patients, albumin levels declined during the acute peri-procedural period (*p* = 0.0002; d = 0.71), with a secondary decline during intermediate recovery (*p* = 0.018; d = 0.51). Levels returned toward baseline during late recovery (181–365 days) and remained relatively stable thereafter. Although overall variation was significant (*p* = 0.0001), the magnitude and persistence of albumin changes were less pronounced than in CABG.

## 4. Discussion

This retrospective, exploratory, longitudinal study analyzed thyroid hormone changes (Free T3, Free T4, and TSH) after cardiac interventions, including AVR, CABG, and PCI. We hypothesized that acute, perioperative disturbances occur and that more invasive surgeries (CABG and AVR) cause greater hormonal shifts. The results suggest a complex link between surgical stress and thyroid function, with important implications for postoperative care.

We found that TSH exhibited a biphasic pattern postoperatively, with an initial elevation during the acute peri-procedural period, followed by normalization or relative suppression by late recovery (181–365 days) in CABG patients. The drop in free T3 levels after CABG was observed early, confirming earlier findings of thyroid axis suppression due to surgical stress [1,10,19]. Free T4 levels were more stable across all procedure groups. The composite picture of the thyroid network aligns with the pathophysiology of non-thyroidal illness syndrome (NTIS), a state in which thyroid hormone abnormalities occur without intrinsic thyroid pathology [19,20,21]. These conditions are common among patients with critical care-grade or cardiovascular illnesses [21]. Observed abnormalities may be an exacerbation of pre-existing thyroid illness, but our data do not allow for longitudinal analysis of thyroid functioning for several years before surgery [21]. Alternatively, a patient may experience euthyroid sick syndrome (EUS) [13]. Interestingly, both EUS and NTIS may represent adaptations rather than pathological deviations, given the significant stress of the peri-surgical period [4,5,7,13,21]. Notably, some of these alterations persisted beyond the early recovery phase, suggesting a potential prolonged association between surgical stress and thyroid axis perturbation. The differential behavior of free T3 and free T4 highlights the role of deiodinase regulation and peripheral conversion under stress, underscoring free T3 as a more responsive biomarker of surgical endocrine impact [7,9,13,20]. These observations are consistent with prior studies indicating that free T4 is less dynamic in acute illness unless the stress is severe or prolonged [21,22]. While some degree of persistence was observed at later timepoints, this likely reflects a combination of biological effects and selective follow-up rather than definitive prolonged dysfunction. Our findings align with those of Klemperer et al., who demonstrated that TSH and T3 abnormalities can persist postoperatively, particularly in critically ill patients [22]. During and after invasive procedures like CABG, elevated dopamine (either endogenous or infused) and a surge in inflammatory cytokines synergize to reduce TRH and TSH signaling [23]. This leads to a blunted or delayed TSH response, even in the presence of low peripheral thyroid hormones. This explains that TSH often remains low-to-normal in the early phases of NTIS and only recovers after systemic inflammation and dopaminergic tone subside [23,24]. However, interpretation of longer-term findings requires caution, given the non-standardized nature of laboratory follow-up. Prolonged suppression of thyroid hormones, particularly in a population with cardiovascular disease, could impair myocardial remodeling, metabolic recovery, and immune function, ultimately attenuating the benefits of surgical intervention [25,26]. These observations may support consideration of thyroid surveillance in selected high-risk patients following CABG, although further prospective studies are required. Patients at risk will have to be identified as NTIS is primarily considered an adaptive and allostatic response, during which thyroid hormone supplementation is not universally recommended [6,7,13,20,26]. However, some evidence supports low-dose T3 therapy in specific contexts, such as cardiac surgery or heart failure, to improve hemodynamics and recovery metrics is an area warranting further investigation [22].

Contrary to one of our primary hypotheses, AVR produced only muted effects on the thyroid axis, despite similar levels of inflammation and stress burden. Concomitant with our hypothesis, PCI, being the least invasive intervention, showed minimal changes in the thyroid axis throughout follow-up at all time points. These findings suggest potential procedure-related differences in endocrine response rather than a simple linear relationship with procedural invasiveness. CABG demonstrated the most consistent early perturbations, although this should not be interpreted as a causal or procedure-specific effect [27]. We can only speculate that intervention on the coronary artery introduces additional factors to explore. The use of antiarrhythmic medication, contrast, or pre-existing comorbidities may also explain the differences observed, but further studies are needed to assess their influence. It is an interesting hypothesis that cardiopulmonary bypass or direct injury to the myocardium may impact the thyroid axis, specifically as it was suggested by some [1,19,21,22,25]. Finally, the effect of preoperative conditions should be factored in during the next iteration of this work, as the degree of myocardial dysfunction is interrelated with the performance of the thyroid axis [7,10,26,28].

This study has several limitations in its secondary applied methodology. We do not know why the patients were tested for thyroid hormones after surgery. The most likely cause is pre-existing thyroid hormone dysfunction, suggesting possible over-representation of patients with thyroid illnesses or taking thyroid supplements. Laboratory measurements were obtained opportunistically as part of routine clinical care rather than through a standardized follow-up protocol. Therefore, laboratory availability at later timepoints likely reflects clinical indications for testing rather than systematic sampling. Patients contributing laboratory data at later timepoints may represent a clinically selected subgroup with greater comorbidity burden or ongoing healthcare utilization, introducing non-random missingness and potential selection bias. Reasons for testing could not be determined in our study. This introduces potential selection bias, particularly for albumin and thyroid measurements obtained several years after the procedure. Consequently, long-term trends should be interpreted cautiously. Likewise, medications known to affect thyroid function, including amiodarone or thyroid hormone replacement therapy, were not consistently available and therefore could not be incorporated into multivariable analyses. Race classification in EMR may not reflect biological ancestry and may introduce classification limitations. Electronic medical records may contain inherited errors and issues.

TSH was often tested post-surgery, likely due to concerns about thyroid function, yet few patients had detailed assessments like T3 or fT3. Our small sample size and lack of baseline data limited the power of the analysis and regression, particularly for some variables. As a retrospective study, causality cannot be confirmed, and confounders such as medications, pre-existing thyroid issues, or nutrition were not fully controlled, especially perioperative medication records. Albumin levels varied preoperatively and are influenced by factors such as liver failure, fluid overload, or social circumstances, making them unpredictable. Hormone measurements lacked consistency across time points, adding selection bias. Large standard deviations, especially in TSH, show considerable inter-individual variation.

Despite significant limitations, some of our study’s results are validated by similar but much shorter observations [1,10,19,22,28]. Sabatino et al. showed that both total and free T3 levels decrease significantly during and after CABG, persisting for several days postoperatively due to proinflammatory cytokines and alterations in deiodinase activity [10,28]. The strengths of this study include extended follow-up beyond the immediate peri-procedural period and inclusion of diverse cardiac interventions. Standardization of laboratory values using Z-scores allowed comparison across different assay platforms and laboratory systems. The use of multiple time points provides a detailed understanding of thyroid hormone recovery, unlike most previous studies, which are limited to perioperative snapshots. However, several limitations must be acknowledged. Therefore, our findings should be regarded as exploratory and hypothesis-forming only.

## 5. Conclusions

In this retrospective exploratory study, CABG was associated with the most pronounced early alterations in thyroid hormone and albumin trajectories, characterized by significant FT3 suppression and sustained albumin decline. AVR and PCI demonstrated more variable and generally less pronounced patterns over time. These findings are consistent with post-procedural metabolic and endocrine stress responses but should be interpreted cautiously given substantial missingness, opportunistic laboratory sampling, and residual confounding. Prospective studies with standardized follow-up are needed to clarify the persistence and clinical significance of these abnormalities.

## Figures and Tables

**Table 1:** Availability of thyroid laboratory measurements at predefined time points.

	Number of Measurements Available		
Timeline (Missing %)	FT3	FT4	TSH
Baseline (8.2%)	113	232	2502
Acute peri-procedural period (51.5%)	69	100	1334
Early recovery (68.9%)	52	98	814
Intermediate recovery (77.9%)	26	75	582
Later recovery (69.5%)	33	109	803
Medium term recovery (61.7%)	34	101	1051
Long-term follow-up recovery (53.5%)	54	133	1253

**Table 2:** Demographics and clinical characteristics of the patients with FT3, FT4, and TSH values.

Variable	AVR	CABG	PCI	p-Value
*Free T3 (AVR n = 44, CABG n = 48, PCI n = 54)*				
Age, mean ± SD	67.5 ± 13.4	66.8 ± 9.5	68.5 ± 14.4	*0.59*
Male (%)	52.3%	60.4%	50.0%	*0.55*
Atrial fibrillation (%)	63.6%	45.8%	25.9%	*0.0009*
Diabetes (%)	27.3%	45.8%	33.3%	*0.16*
Hypertension (%)	27.3%	45.8%	31.5%	*0.14*
White (%)	72.7%	66.7%	68.5%	*0.85*
African American (%)	20.5%	20.8%	24.1%	*0.85*
Asian (%)	2.3%	6.2%	1.9%	*0.85*
Other (%)	4.5%	6.2%	5.6%	*0.85*
***Free T4 (AVR n = 84, CABG n = 92, PCI n = 106)***				
Age, mean ± SD	64.4 ± 12.5	66.5 ± 9.2	66.6 ± 14.1	*0.37*
Male sex (%)	57.1%	71.7%	53.8%	*0.01*
Atrial fibrillation (%)	61.9%	47.8%	20.8%	*<0.001*
Diabetes mellitus (%)	28.6%	41.3%	34.9%	*0.16*
Hypertension (%)	35.7%	47.8%	34.9%	*0.11*
White (%)	75.0%	67.4%	58.5%	*0.04*
Black or African American (%)	11.9%	19.6%	34.0%	*<0.001*
Asian (%)	2.4%	5.4%	0.9%	*0.29*
Other (%)	10.7%	7.6%	6.6%	*0.58*
***TSH (AVR n = 681, CABG n = 824, PCI n = 1170)***				
Age, mean ± SD	65.4 ± 12.0	66.5 ± 9.5	66.2 ± 12.6	*0.18*
Male sex (%)	66.8%	74.4%	63.2%	*<0.001*
Atrial fibrillation (%)	58.3%	43.2%	17.6%	*<0.001*
Diabetes mellitus (%)	26.6%	46.5%	35.2%	*<0.001*
Hypertension (%)	37.3%	49.5%	40.6%	*<0.001*
White (%)	74.3%	69.2%	58.1%	*<0.001*
Black or African American (%)	12.5%	16.7%	30.6%	*<0.001*
Asian (%)	2.8%	2.8%	2.4%	*0.87*
Other (%)	10.4%	11.3%	8.9%	*0.41*

**Demographic tables:** Continuous variables are presented as mean ± standard deviation and compared using ANOVA or Kruskal–Wallis tests as appropriate. Categorical variables are expressed as percentages and compared using the chi-square test. Race categories represent classifications recorded in the electronic medical record.

## Data Availability

The original contributions presented in this study are included in the article. Further inquiries can be directed to the corresponding author.

## Abbreviations

The following abbreviations are used in this manuscript:

AVR	Aortic Valve Replacement
CABG	Coronary Artery Bypass Graft
PCI	Percutaneous Coronary Intervention
EMR	Electronic Medical Records
FT3/fT3	Free Triiodothyronine
FT4/fT4	Free Thyroxine
TSH	Thyroid-Stimulating Hormone
NTIS	Non-Thyroidal Illness Syndrome
EUS	Euthyroid Sick Syndrome
TRH	Thyrotropin-Releasing Hormone
SD	Standard Deviation
IQR	Interquartile Range
d (Cohen’s d)	Effect Size (Cohen’s d)
ICD-10	International Classification of Diseases, 10th Revision
UPenn	University of Pennsylvania
Python	High-level Programming Language Used for Data Processing and Analysis
Seaborn	Data Visualization Library in Python
SciPy	Python-Based Ecosystem for Scientific and Mathematical Computing
Pandas	Data Analysis and Manipulation Library in Python
Matplotlib	Python 2D Plotting Library
